# Hypertension Improvement Project (HIP): study protocol and implementation challenges

**DOI:** 10.1186/1745-6215-10-13

**Published:** 2009-02-26

**Authors:** Rowena J Dolor, William S Yancy, William F Owen, David B Matchar, Gregory P Samsa, Kathryn I Pollak, Pao-Hwa Lin, Jamy D Ard, Maxwell Prempeh, Heather L McGuire, Bryan C Batch, William Fan, Laura P Svetkey

**Affiliations:** 1 Department of Medicine, Duke University Medical Center, Durham, NC, USA; 2 Center for Health Services Research in Primary Care, Veterans Affairs Medical Center, Durham, NC, USA; 3 President's Office, University of Medicine and Dentistry of New Jersey, Newark, NJ, USA; 4 Center for Clinical Health Policy Research, Duke University Medical Center, Durham, NC, USA; 5 Department of Biostatistics & Bioinformatics, Duke University, Durham, NC, USA; 6 Department of Community and Family Medicine, Duke University Medical Center, Durham, NC, USA; 7 Cancer Prevention, Detection, and Control Research Program, Duke University Medical Center, Durham, NC, USA; 8 Nutrition Sciences, University of Alabama, Birmingham, AL, USA; 9 Department of Nephrology, Billings Clinic, Billings, MT, USA; 10 Cary Kidney Center, Cary, NC, USA; 11 Duke Hypertension Center, Duke University Medical Center, Durham, NC, USA; 12 Sarah W Stedman Nutrition and Metabolism Center, Duke University Medical Center, Durham, NC, USA

## Abstract

**Background:**

Hypertension affects 29% of the adult U.S. population and is a leading cause of heart disease, stroke, and kidney failure. Despite numerous effective treatments, only 53% of people with hypertension are at goal blood pressure. The chronic care model suggests that blood pressure control can be achieved by improving how patients and physicians address patient self-care.

**Methods and design:**

This paper describes the protocol of a nested 2 × 2 randomized controlled trial to test the separate and combined effects on systolic blood pressure of a behavioral intervention for patients and a quality improvement-type intervention for physicians. Primary care practices were randomly assigned to the physician intervention or to the physician control condition. Physician randomization occurred at the clinic level. The physician intervention included training and performance monitoring. The training comprised 2 internet-based modules detailing both the JNC-7 hypertension guidelines and lifestyle modifications for hypertension. Performance data were collected for 18 months, and feedback was provided to physicians every 3 months. Patient participants in both intervention and control clinics were individually randomized to the patient intervention or to usual care. The patient intervention consisted of a 6-month behavioral intervention conducted by trained interventionists in 20 group sessions, followed by 12 monthly phone contacts by community health advisors. Follow-up measurements were performed at 6 and 18 months. The primary outcome was the mean change in systolic blood pressure at 6 months. Secondary outcomes were diastolic blood pressure and the proportion of patients with adequate blood pressure control at 6 and 18 months.

**Discussion:**

Overall, 8 practices (4 per treatment group), 32 physicians (4 per practice; 16 per treatment group), and 574 patients (289 control and 285 intervention) were enrolled. Baseline characteristics of patients and providers and the challenges faced during study implementation are presented. The HIP interventions may improve blood pressure control and lower cardiovascular disease risk in a primary care practice setting by addressing key components of the chronic care model. The study design allows an assessment of the effectiveness and cost of physician and patient interventions separately, so that health care organizations can make informed decisions about implementation of 1 or both interventions in the context of local resources.

**Trial registration:**

ClinicalTrials.gov identifier NCT00201136

## Background

Approximately 50 million adult Americans have hypertension [1], which is the most prevalent risk factor for cardiovascular and kidney disease [2] and accounts for approximately 35% of atherosclerotic cardiovascular disease (CVD) [3]. Fortunately, the treatment of hypertension reduces associated risks: lowering blood pressure (BP) by a variety of strategies reduces the risk of stroke by approximately 35%, congestive heart failure by 42%, and coronary heart disease by 28% [4-7]. Therefore, an important key to reducing the burden of hypertension-related CVD is to increase the proportion of patients who achieve optimal BP control. To promote this goal, the Joint National Committee on Prevention, Evaluation, Detection, and Treatment of High Blood Pressure (JNC) has established guidelines that have been widely disseminated to practitioners through the National Institutes of Health High Blood Pressure Education Program and other organizations [8].

The latest JNC guidelines (JNC-7), published in 2003, classify BP into stages and provide recommendations for treatment and follow-up. The recommended goal for patients with uncomplicated hypertension is an average systolic BP of less than 140 mm Hg and a diastolic BP of less than 90 mm Hg. For patients with certain comorbid conditions that increase risk (i.e., diabetes mellitus and chronic kidney disease), the goal BP is less than 130/80 mm Hg [8].

Treatments for high blood pressure are classified as lifestyle modification or medication. The guidelines direct physicians to prescribe lifestyle modification to all patients with BP that is above the optimal category (> 120/80 mm Hg). The recommendations for lifestyle modification are evidence-based and include adoption of a healthy dietary pattern such as the Dietary Approaches to Stop Hypertension (DASH) diet [9], losing weight if overweight, reducing sodium intake, increasing physical activity, and limiting alcohol intake [8]. Each of these behavioral interventions has been shown to lower BP and contribute to hypertension control. For medication therapy, the guidelines indicate when and whom to treat and also provide advice on choice of drug, when and how to adjust doses, and how to minimize non-adherence to therapy. The explicit intent of the guidelines is to assist physicians in achieving goal BP in the majority of their patients.

Two behaviors that could improve BP control are physicians' adherence to JNC-7 guidelines and patients' adherence to lifestyle recommendations and medication regimens. Several similarities exist between behavioral models that attempt to explain why physicians do or do not follow guidelines for preventive care [10,11] and those that attempt to explain why patients do or do not adopt healthy lifestyle behaviors [12,13]. For the physician, awareness (knowledge), motivation, and confidence (self-efficacy) all influence adherence to guidelines. For instance, physicians who do not know about or do not feel motivated to follow the JNC guidelines are not likely to prescribe them, nor are physicians who have the knowledge and motivation but lack confidence to follow the guidelines. Non-behavioral factors can also influence physicians' actions. These include external or systems-level factors such as time constraints, resources, and systems to prompt or remind physicians of the guidelines [10,11,14].

**Figure 1:**
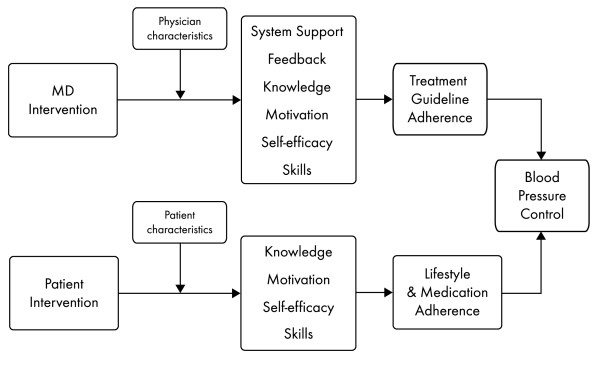
**Psychosocial mediators and systems factors that influence physician and patient adherence to JNC-7 guidelines**. MD = physician.

Similarly, behavioral factors may also influence patients' adherence to lifestyle recommendations and medications [12,13]. Patients who are unaware that they need to change their behavior or who are not motivated will not change. In addition, external factors including food availability and environmental structures may affect how patients adhere to a healthy lifestyle. The Hypertension Improvement Project (HIP) behavioral interventions were designed to target the same psychosocial mediators and systems factors for both physicians and patients, presented graphically in Figure 1.

Strategies for implementing recommended practice to improve clinical outcomes include dissemination of printed educational materials (e.g., clinical practice guidelines), physician and patient training, feedback to physicians, dissemination of information linked to performance (e.g., audit and feedback, reminder systems), clinical performance measures (CPMs), and simple clinical bedside tools. Although randomized trials of these strategies are rare, there is evidence that such tools can change medical provider behavior and improve clinical outcomes [15-17]. A major challenge is the integration of practice improvement strategies into the flow of clinical care; to accomplish this, health researchers and providers have borrowed techniques of Continuous Quality Improvement (CQI). It is the application of a CQI approach–applying tools shown to be effective–that forms the foundation for the HIP physician intervention.

The patient also plays a critical role in hypertension care. As noted above, the JNC guidelines include lifestyle modification for all patients with hypertension. Programs that result in successful lifestyle behavior change are generally based on Social Cognitive Theory [12] and techniques of behavioral self-management [18]. They are typically constructed using the Transtheoretical Model [13,19] and motivational enhancement approaches [20,21]. These approaches emphasize the importance of the individual's ability to regulate behavior by setting goals, developing specific behavior change plans, monitoring progress towards the goals, and attaining skills necessary to reach the goals. Self-efficacy (i.e., one's confidence in performing a given behavior) and outcome expectations (i.e., one's belief that changing behavior leads to a favorable outcome) are critical mediators of behavior change [12,22]. An example of this approach can be found in the PREMIER trial of lifestyle interventions for blood pressure control–a multi-center trial sponsored by the National Heart, Lung, and Blood Institute that tested the effects on BP of 2 multi-component lifestyle interventions, relative to an advice-only control condition [23]. The PREMIER study demonstrated that a 6-month intensive behavioral intervention leads to healthy lifestyle changes, significant reductions in BP, and improved control of hypertension.

As is true for most chronic illnesses, hypertension management requires extensive efforts from physicians and patients alike, with support from the health care system. It was hypothesized that a quality improvement approach would maximize physician adherence to treatment guidelines, and a behavioral intervention would improve patient adherence to lifestyle recommendations. Both approaches may lead to significant improvements in BP control with consequent reductions in CVD risk. The HIP study offers a unique and innovative opportunity to test this hypothesis by determining, in a randomized controlled trial, the separate and combined effect on BP of a physician intervention and a patient intervention. The study design provides for an assessment of the effectiveness and cost of implementing the physician and patient interventions separately, thereby allowing health care organizations to make informed decisions about implementation of 1 or both interventions in the context of local resources. As participant enrollment concludes and data cleaning and analysis begin, this paper describes the protocol of the HIP trial and discusses the lessons learned thus far from designing and implementing the study, including issues encountered and subsequent changes to the study design.

## Methods and design

### Overview

**Figure 2:**
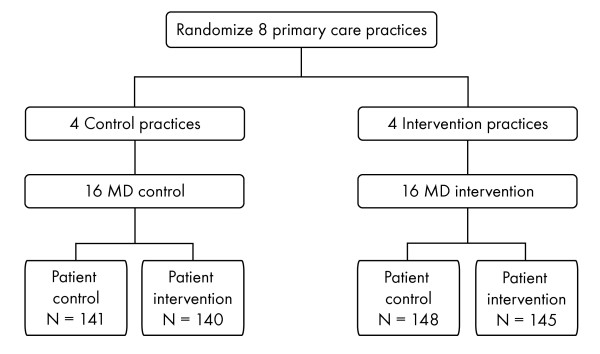
**Design of the HIP study**. MD = physician.

The HIP study was a nested 2 × 2 randomized controlled trial of a physician intervention, a patient intervention, and both combined (Figure 2). All study procedures were approved by the Duke Institutional Review Board. Recruitment was conducted in waves: i.e., 2 practices were initiated every 6 months to allow adequate time for study personnel to train providers and recruit participants. Each group of practices was considered a cohort (e.g., the first 2 practices recruited are considered Cohort 1). Nesting occurred at the level of the practice and the level of the physician. Primary care practices were randomly assigned to the physician (MD) intervention or to the MD control condition. All participating MDs within a given practice had the same randomization assignment. The MD intervention consisted of training and performance monitoring. Performance data were collected for 18 months, and feedback was provided to physicians every 3 months within that timeframe.

Within the practices, patient participants were individually randomized to the patient intervention or to usual care. The patient intervention occurred shortly after initiation of the physician intervention and consisted of a 6-month group-based behavioral intervention conducted by trained interventionists, followed by brief monthly phone counseling for 12 months from a community health advisor (CHA). Follow-up measurements were performed at 6 and 18 months following randomization.

### Enrollment and randomization of MDs

To avoid contamination among physicians in the same practice, randomization occurred at the level of the practice. Four matched pairs of primary care practices were randomly assigned to physician intervention or control. Practices were matched according to specialty (internal medicine or family practice) and participant mix (percent of participants with Medicaid or Medicare without supplemental insurance). Within each practice, all physicians were invited to participate, with a goal of enrolling 4 physicians at each clinic. All physicians at a given clinic were assigned to the same intervention.

### Enrollment and randomization of patients

After enrolling clinics and physicians, participants were then recruited from the patients of the enrolled physicians. Ten to 15 patients cared for by each participating MD were enrolled and randomized to the behavioral intervention or usual care.

Billing codes were used to identify hypertensive patients. Each participating physician reviewed the list and removed names of patients considered unsuitable candidates for study participation. Recruitment letters were mailed on practice letterhead with the physician's name on the signature line. Patients were asked to call the research coordinator within 10 working days to decline participation in the study. After the 10 days, a member from the study staff called unresponsive patients to assess their willingness and eligibility to participate (Table 1).

**Table 1:** HIP eligibility criteria

**Inclusion criteria**	• Receiving primary care from a randomized physician
	• Diagnosis of hypertension: Average SBP ≥ 140 mm Hg and/or DBP ≥ 90 mm Hg on at least 2 clinic visits in the past 12 months or taking antihypertensive medication
	• Age 25 years or older at the time of the initial screening visit
	• Willing and able to participate fully in all aspects of the intervention
**Exclusion criteria**	• Cardiovascular event within the past 6 months (if more remote history, eligible with MD approval)
	• Chronic kidney disease (estimated GFR < 60 ml/min)
	• Planning to leave the area prior to the anticipated end of participation
	• Pregnant, breast feeding, or planning pregnancy prior to the end of participation
	• Current participation in another clinical trial
	• Investigator discretion for safety or adherence reasons
	• Household member of another HIP participant or of an HIP staff member

DBP = diastolic blood pressure; GFR = glomerular filtration rate; HIP = Hypertension Improvement Project; MD = physician; SBP = systolic blood pressure.

At the first screening visit, the consent form was reviewed and signed, height, weight and BP were measured, and body mass index (BMI) was calculated. A self-administered medical history questionnaire was used to establish the final set of eligibility criteria. At a second screening visit, questionnaires and interviews established the patient's commitment and ability to participate, and a fasting blood specimen, 24-hour urine collection, and a second BP measurement were obtained. Participant baseline characteristics are shown in Table 2.

**Table 2:** Baseline patient characteristics (N = 574)

**Characteristic**	%*
N	574

Age, mean years (SD)	60.5 (11.4)

Female sex	61

Completed high school	93

Adequate income (self-reported)	85

African-American race	37

Medical history	

Taking BP medications	95

Ever smoked	48

Physical activity (accelerometry), mean minutes/week (SD)	34 (106)

Diabetes	30

Hyperlipidemia	48

* Unless otherwise indicated.

BP = blood pressure; SD = standard deviation.

After eligibility was established, randomization occurred using a computer-generated algorithm, stratified by cohort and by clinic. Participants were randomized in varying block sizes to ensure comparable numbers of participants in each assignment over time. Each participant learned of his/her treatment assignment from a HIP staff member who was not involved in collecting study measurements. Intervention staff members then instructed participants on the details of their assigned intervention.

### Content and conduct of physician intervention

The MD control constituted a "usual care" condition. That is, there was no attempt to change or monitor whatever procedures were already in place in that practice for quality improvement and physician education with regard to BP control. No performance data were collected from these physicians, and no performance feedback was given.

The active MD intervention consisted of 3 main elements. The first element included 2 training modules provided as on-line continuing medical education (CME) courses. The first module addressed the JNC-7 guidelines and the second addressed lifestyle modification for BP control. The modules were available in 3 formats (streaming audio, PowerPoint slide show, and print version) so that each physician could choose the most convenient modality. Each module required approximately 45 minutes to complete, included a quiz that gave immediate feedback on the answers, and resulted in CME credit through the Duke CME office. All physicians completed the modules before the first group session of the patient intervention.

The second element was an evaluation and treatment algorithm that summarized the major guidelines set forth in JNC-7. The BP management algorithm–derived from the JNC-7 guidelines and formatted as a decision tree–was mass-produced in a color-coded, laminated pocket card (Additional file 1) and provided to each physician randomized to the active MD intervention. The algorithm directly informed the portion of the CPM data form that was completed by the MD (Additional file 2).

The third element was a CQI-type procedure involving assessment of CPMs and quarterly feedback to physicians on their adherence to guidelines, including those related to lifestyle counseling. At each clinic randomized to the MD intervention, the clinic staff and participating physician completed a single-page CPM data form every time an HIP participant (in either patient treatment group) had a clinic visit. A clinic nurse completed the top half of the form (BP at current and last clinic visit, comorbidities), and the MD completed the bottom half (current BP medications, actions taken during the visit, follow-up interval). In addition, data from non-study participants were collected on anonymous forms 1 day each month to increase sample sizes for feedback reports. In both cases, the form was attached to the billing encounter form to prompt MDs to complete it. These data were converted into quarterly feedback reports that indicated to each physician: 1) the proportion of hypertensive participants with adequately controlled BP for that quarter and the previous quarters; 2) the proportion not at goal who had medication adjustments at the visit; 3) the proportion that received lifestyle modification counseling; 4) the proportion without diabetes (DM) or chronic kidney disease (CKD) who were at goal BP and also the proportion prescribed a thiazide diuretic; 5) the proportion with DM and/or CKD who were at goal BP and also the proportion prescribed an angiotensin-converting enzyme inhibitor/angiotensin receptor blocker; and 6) comparisons with peer MDs for each category.

### Content and conduct of the patient intervention

#### Patient control

Participants randomized to the control group had an individual brief visit with an interventionist after randomization, during which they received advice and brochures on lifestyle modification for BP control consistent with JNC-7. At the end of the study (18 months), after the final data collection visit, participants in the control group were offered an abbreviated version of the active intervention, which consisted of 6 weekly group sessions to help them make lifestyle changes to control BP (wait-list control).

#### Active intervention: structure and content

The active patient intervention was based on key theoretical constructs developed to guide health behavior change efforts, and on practical applications from previous trials of lifestyle change and CVD risk reduction [24-26].

The patient intervention was conducted by 2 interventionists who served in this role in previous studies. Training for the interventionists was provided by behavioral scientists and nutritionists, and included training in intervention content, group facilitation, and motivational interviewing [20]. Ongoing quality assurance for intervention delivery was provided through retraining, observation, and feedback for the interventionists. The patient intervention consisted of 20 weekly sessions provided over approximately 6 months, during which the participants met in small groups (n = 10–15 per group) with an interventionist. All intervention sessions took place at or near the participants' clinic site, providing a familiar location for these sessions.

Specific behavior change strategies comprising the HIP patient intervention are listed in Table 3. The materials developed to deliver the patient intervention included a leader's guide and a participant manual featuring self-monitoring tools. The leader's guide provided a standardized framework and structure for each group session, as well as resource materials for session discussions. The participant manual provided the general format, outline, and worksheets for each session. It included information about diet and physical activity, and emphasized changing behaviors. This manual was intended to complement the group session process and content by serving as a workbook during the sessions and as a reference between sessions.

**Table 3:** Behavioral change strategies of the HIP active intervention

**Strategy**	**Definition**
Frequent contact	Attend weekly sessions for 20 weeks.

Group interaction and social support	Sessions were highly interactive and minimally didactic; participants were encouraged to share experiences that led to patient modeling behavior; they also were encouraged to help each other solve problems.

Goal setting and self-monitoring	Emphasis placed on individual's ability to regulate his/her behavior by setting goals and monitoring progress towards the goals. Participants kept records of dietary intake, physical activity, and medication usage at least 3 days a week. Records were reviewed by the interventionist to provide feedback and encourage or support participant's behavior change.

Identification of barriers and problem-solving	Interventions were patient-centered; interventionist assisted participant in identifying his/her own barriers and generating solutions.

Motivational interviewing	Patient-centered counseling emphasized support of self-efficacy and optimism for change; included reflective listening, objective feedback, and respect.

HIP = Hypertension Improvement Project.

The study also employed volunteer community health advisors (CHAs) who were identified and recruited from the same communities as the target population. These "natural leaders" were trained to participate in all aspects of the patient intervention. They assisted the interventionists in conducting the group sessions and were asked to lead portions of the activities or discussion so that the intervention was delivered in conjunction with members of the community. The CHAs also made monthly calls during the 6 months while the intervention was delivered and during the 12 months after the intervention. The purpose of these calls was to provide one-on-one counseling to encourage participants to make or maintain behavior changes. The above-mentioned leader's guide was developed at the appropriate education level and with cultural sensitivity so that it could also be used to train the CHAs. The CHAs participated in a 20-hour training program consisting of 4 weekly sessions covering general information about hypertension, dietary and physical activity interventions for BP control, community resources, facilitation of group education sessions, practical guidelines for helping peers, listening skills, lifestyle behavior change techniques, and skills in stress management, problem-solving, and goal setting. Certification and on-going supervision were similar to that provided to the interventionists.

The CHAs served as familiar, non-authoritative resources for study participants, strengthening communication between the research group and the participants and providing additional social support. The intent is that the CHAs will have sufficient knowledge and skills to serve as ongoing resources in their communities and to help sustain the effects of the project after it is completed.

The outcomes were measured at the level of the patient, who was exposed to neither intervention, MD intervention alone, patient intervention alone, or both interventions. Overall, 8 practices (4 per treatment group), 32 physicians (4 per practice; 16 per treatment group), and 574 patients (289 control and 285 intervention) were enrolled (Figure 2).

### Physician measurements

Measurements specific to the physician intervention are described above. All physicians, regardless of treatment assignment, were asked to complete a baseline questionnaire concerning their demographics, education, and training–variables that are potential mediators of the effect of the physician intervention on BP control (Table 4). In addition, at entry, 6 months, and 18 months, all physicians were asked to complete a questionnaire in which they reported their practice patterns and habits. For example, providers reported the average amount of time spent discussing hypertension and lifestyle changes with patients, in addition to addressing barriers to, confidence in, and patient adherence to lifestyle modification counseling.

**Table 4:** Baseline physician characteristics (N = 32)

**Characteristic**	%*
Age, mean years (SD)	47.9 (9.9)

Female sex	34

African-American race	16

Family medicine specialty	53

Internal medicine specialty	47

Years since MD degree, mean (SD)	20.7 (10.0)

Patients with HTN	28.9

Very familiar with JNC guidelines	31

* Unless otherwise indicated.

HTN = hypertension; JNC = Joint National Committee on Prevention, Evaluation, Detection, and Treatment of High Blood Pressure; SD = standard deviation.

### Patient measurements

All study measurements obtained from participants were collected during face-to-face clinic visits by trained, certified study personnel who were blinded to intervention assignment. At each time point (baseline, 6- and 18-month follow-up), BP was measured according to JNC-7 guidelines (i.e., seated quietly with back supported and feet on the floor for 5 minutes prior, appropriate size cuff, arm bared and supported, no ingestion or smoking for 30 minutes prior) by an oscillometric blood pressure machine. At each study visit, average BP was defined as the averaging of duplicate measurements taken on each of 2 separate visits at least 1 week apart. BP control was defined based on JNC-7 guidelines: average systolic BP < 140 mm Hg and diastolic BP < 90 mm Hg for participants without renal disease or diabetes, and systolic BP < 130 mm Hg and diastolic BP < 80 mm Hg for participants with 1 or both of these conditions. Other measurements included weight/BMI, a dietary assessment using the Block Food Frequency Questionnaire [27], a blood sample obtained after an overnight fast, a 24-hour urine collection, an objective assessment of physical activity using a triaxial accelerometer [28], an assessment of medication adherence by self report, and psychosocial questionnaires. The measurement schedules for patients and physicians are shown in Table 5.

**Table 5:** Measurement schedule

**Time/variable**	**Baseline**	**3 mo.**	**6 mo.**	**9 mo.**	**12 mo.**	**15 mo.**	**18 mo.**
**Physicians**							

Personal characteristics and training	**X**						

Practice habits (self-report)	**X**		**x**				**x**

Clinical performance measure (intervention group only)	**X**	**x**	**x**	**x**	**x**	**x**	**x**

**Participants**							

Blood pressure (Omron HEM-907, average of 4 readings at 2 visits 1 week apart)	**X**		**x**				**x**

Weight	**X**		**x**				**x**

Fasting lipid panel	**X**		**x**				**x**

24-Hour urine collection	**X**		**x**				**x**

Diet (Block food frequency questionnaire)	**X**		**x**				**x**

Physical activity (7-day physical activity recall)	**X**		**x**				**x**

Physical activity (triaxial accelerometer)	**X**		**x**				**x**

Medication adherence (self-reported medication-taking scale)	**X**		**x**				**x**

Psychosocial mediators (SF-36, social support, perceived stress, depression)	**X**		**x**				**x**

Symptom questionnaire	**X**		**x**				**x**

Medication questionnaire	**X**		**x**				**x**

Process measures (patient intervention only: attendance, self-monitoring records)	**X**	**x**	**x**				

### Primary and secondary outcomes

The primary outcome was the change in systolic BP at 6 months. Secondary outcomes included change in diastolic BP, the proportion of participants with adequate BP control at 6 and 18 months, and the effect of treatment on behavior change (i.e., weight loss, dietary pattern, physical activity) at 6 and 18 months. The 6-month outcomes correspond to the maximum impact of the patient intervention but may be too early to reflect maximum impact of the MD intervention. The 18-month outcomes assess the durability of the patient intervention and the cumulative (perhaps maximal) effect of the MD intervention.

### Analysis plan

The data cleaning and analysis stage of the HIP trial has begun. The adequacy of randomization will be assessed by comparing the baseline characteristics of the 4 study groups. Chi-square tests will be used for categorical variables, and the analysis of variance will be used for continuous variables.

For the patient intervention groups only, various measures pertaining to adherence to the patient intervention and its impact on intermediate outcomes will be summarized. For example, for each cohort, the number of intervention sessions attended will be summarized. Similarly, changes in weight, diet, and exercise will be tabulated, and whether the mean changes equal zero will be determined.

For the main analysis of BP changes, results by group and cohort will first be summarized, and then tested for a cohort-by-group interaction. If this interaction is non-significant–suggesting that the intervention effects are similar across cohorts–summary results will be reported but will include cohort as a control variable in all models. For the continuous outcome of systolic BP, the main comparison will be derived from an analysis of covariance model that controls for cohort and baseline systolic BP. The primary hypotheses of interest will be the main effect of the patient intervention, the main effect of the physician intervention, and the patient-by-physician interaction. This last interaction assesses, for example, whether the patient and physician interventions have a separate impact on outcome or, alternatively, whether they act synergistically. For the categorical outcome of percentage of patients at goal, a similar strategy will be followed using logistic regression, and these analyses will also be stratified by whether the patient was at goal at baseline.

The analysis plan accounts for the correlations among the responses for individual patients, these correlations being induced by the nesting of time points within patients within physicians within practices. The analysis of variance paradigm will be used, accounting for the correlations among the 3 time points by separately analyzing the paired scores representing differences between baseline and 6 months and between baseline and 18 months. These comparisons can be preceded by a statistical test, such as Hotelling's T^2^, that takes explicit account of these correlations.

### Sample size and statistical power

The power calculations assumed that the impact of accounting for covariates such as baseline blood pressure (which increases statistical power) and the impact of the clustering inherent to this design (which decreases statistical power) would approximately offset and, thus, that power calculations from the 1-way analysis of variance would apply. If so, having approximately 340 patients complete the study would yield approximately 80% power to detect main effects, although the power to detect an interaction between the physician intervention and patient intervention would be smaller. After accounting for expected drop-outs, the minimum sample size for the primary analysis was set at 400.

Preliminary planning also considered 2 other factors, both of which supported an increase in the sample size above 400, if feasible. First, it was anticipated that the study would analyze various secondary outcome variables, some of which might have effect sizes that were less than 0.3. Second, so that the results could be as generalizable as possible, the study strove to include as many clinics as possible. The design originally intended to include 5 sets of clinics, a number which was also suggested by the fact that the most conservative statistical analysis of the impact of the physician intervention–namely, a sign test that simply counts which clinic out of each pair had the better outcome–would be statistically significant if the physician intervention outperformed the controls at each pair of clinics.

Near the conclusion of the 6-month follow-up for Cohort 3, the Data Safety Monitoring Board (DSMB) reviewed the study design–in particular, whether it would, in view of the trial's limited budget, be appropriate to reduce the number of cohorts from 5 to 4. This DSMB review included a comparison of the results of the study to date (to which the investigators, with the exception of the study statistician, were blinded) with the assumptions of the power calculations. In approximate numbers, instead of 40% of patients maintaining BP control at baseline, the figure was actually 70%. Moreover, over 95% of the patients who were in BP control at baseline were also in control at 6 months, raising the possibility that using a dichotomous BP control variable as the primary outcome would suffer from a ceiling effect. (Note that such a ceiling effect was not necessarily certain; for example, an effect size of 0.3 could still be generated from a pattern of outcomes centered around 70%, but this preliminary analysis did suggest that patients who were in BP control at baseline were likely to provide less information than desired toward distinguishing the impacts of the interventions.)

In light of these considerations, the DSMB recommended: (a) to reduce the number of cohorts to 4 (to conserve budget); (b) to focus recruitment in Cohort 4 on patients who were not in BP control at baseline (to ensure that the study population approximated the original target population as much as possible); and (c) to change the primary outcome to change in SBP, measured as a continuous variable, while maintaining the dichotomous measure of BP control as a secondary outcome (to increase statistical power by, for example, being able to differentiate between greater decreases in SBP among patients who were in BP control at baseline).

### Cost analysis

The benefits of the proposed interventions must be weighed in the context of the extent to which they can be delivered systematically to large populations. Thus, the logistical barriers and the actual costs of implementation must be determined as part of an assessment of the value of the HIP interventions. Ultimately, the implementation costs of the patient and physician interventions can be compared with each other and with other approaches to improving BP control. Therefore, a cost analysis will be performed to assess the direct costs of implementation, accounting for both fixed and variable costs for start-up and maintenance.

## Discussion

The chronic care model suggests that BP control can be achieved by improving patient self-care and the systems through which care is delivered [29-32]. Patient self-care efforts should be directed at counteracting the effects on BP of obesity, physical inactivity, poor dietary pattern, and non-adherence to prescribed medications [33]. Furthermore, efforts should be made to promote the use of quality improvement systems that can increase physician adherence to established clinical practice guidelines [34,35]. In addition to improving BP control, these approaches can also reduce costs associated with hypertension and its consequences [29]. However, given the resources required to implement such approaches, it is critical that their effectiveness be rigorously established [36].

The Hypertension Improvement Project is a controlled trial to test the separate and combined effects on BP of a behavioral intervention for patients and a quality improvement intervention for physicians. The patient intervention employed proven behavioral methods for promoting healthy lifestyle and adherence to medication regimens. The physician intervention used a quality improvement approach to provide training, motivation, and feedback on performance in a non-threatening way to promote continuous self-improvement and adherence to clinical practice guidelines.

The physician intervention represents a form of quality improvement that can be described as "Facilitated Process Improvement" (FPI) [37]. Similar to CQI, FPI recognizes that a key barrier to implementing systematic improvement programs is provider time: time to become expert in and to apply CQI methodology and time to become familiar with the sorts of interventions that are likely to be effective in a given context. In FPI, several components of the CQI process are delegated to an expert team, with concurrent agreement by providers during education programs. In this case, the team identified the common limiting barriers to optimal outcomes–lack of easy access to guidelines and lack of ongoing feedback regarding conformance to guidelines (the latter issue may be termed "the difficulty of looking over one's own shoulder")–and created tools to address those barriers.

At the time at which this manuscript was prepared, the trial was in progress. All physicians and patients had been enrolled, and experience with the logistical aspects of study conduct had been garnered. There are numerous unique features and lessons gleaned thus far from the conduct of the trial that will set the stage for interpretation of the study results. More importantly, these features and lessons can inform other investigators planning similar studies.

Several of the challenges faced relate to the setting in which the trial is being conducted (Table 6). The study team considered it important to conduct the study in community clinics where such an approach to BP control would ultimately be implemented. Implementation of the HIP trial within a practice-based research network, while innovative, also required attention to issues that are not usually encountered within an academic medical center (AMC). The study required access to work space that was convenient and familiar to participants (i.e., in or near the clinic) but that had minimal impact on clinical care delivery. Not all medical clinics have space for study visits and group intervention sessions. The study team worked closely with clinic staff to schedule study visits on days when clinic rooms were available (e.g., provider day off) and used the waiting room (in the evenings), the clinic conference room, or a nearby facility for the group sessions.

**Table 6:** Study implementation issues

**Challenge**	**Solution**
**Setting**	

Access to clinic space for study visits	Schedule study visits on days when clinic rooms are available.

Access to space for group intervention	Schedule evening sessions; find local facility.

Minimize disruption to practice flow	Study staff responsible for scheduling, check-in, and posting of signage.

Engage clinic staff/communication	Group meeting prior to (and early part) of implementation; use of e-mail; financial compensation to offset practice costs.

Travel to distant sites	Staff coordinate travel together to minimize travel costs.

**Patient population/recruitment**	

Enroll low SES population	Target participants with no insurance, with Medicaid or Medicare without supplemental insurance.

Identify minority population	Target clinics with more minority participants.

Recruit men to participate	Prioritize mailings and phone calls to men.

High proportion of participants at BP goal (clinics are doing well in BP management; volunteer bias)	Chart review to identify patients with uncontrolled BP prior to screening for Cohort 4.

**Group intervention**	

Finding patients motivated to undergo group intervention	Recruitment by opt-out changed to opt-in/opt-out method.

Attendance	Useful information; mimics real-world implementation.

No individualized counseling (allows implementation in busy setting)	Use community health advisors.

**Physician intervention**	

MD turnover	Refer participants to another participating provider within practice.

Additional work for CPM form completion	Form on non-carbon paper to use as clinic note; brief 1-page form; top part can be filled out by non-MD.

MD time for training module completion	Face-to-face orientation of providers to Web-based (asynchronous) training modules; CME credits offered.

QI developed by researchers (non-practice staff); competing QI initiatives within practice	In future, increase involvement of practice clinicians; use of electronic records to collect BP at routine visits.

Town-gown relationship	Work with existing research partner (PCRC).

BP = blood pressure; CME = continuing medical education; CPM = clinical performance measure; QI = Quality Improvement; JNC = Joint National Committee on Prevention, Evaluation, Detection, and Treatment of High Blood Pressure; MD = physician; PCRC = Primary Care Research Consortium; SES = socioeconomic status.

Even though the study strived to cause minimal disruption to the usual clinic flow, the study team relied on the assistance of clinic personnel to arrange and facilitate several study processes. For example, clinic staff directed participants to the appropriate location for study visits, provided phlebotomy services, distributed blank CPM forms, and collected completed CPM forms. Each of these activities added to the staff's usual responsibilities, but several approaches helped to promote cooperation among study personnel and clinic personnel. First, the study goals and procedures were explained to clinic personnel prior to start-up of a new site. During enrollment and follow-up, the study team maintained frequent and open communication among study coordinators, investigators, and clinic management. Lastly, the study budget included modest compensation to the practices and to the enrolled physicians.

Travel from the AMC to the distant sites (ranging 10–40 miles) was not a trivial issue. The study team tried to travel as a group to minimize costs, and travel expenses were reimbursed. Rising fuel costs during the study affected the budget significantly.

Other challenges related to the choice of study population. The initial goal was to recruit low-income, ethnic minority participants, who are well-represented in the communities served by the participating clinic network. The study also aimed to comprise a population that was approximately 40% male, as men are often under-represented in lifestyle intervention studies. Because participants were recruited from the practices of enrolled MDs rather than the local community, previously effective targeted recruitment procedures could not be relied upon [38]. However, recruitment was targeted through access to the clinical database serving the participating practices, in which race, insurance, and sex data were available. Enrollment of low-income participants was encouraged by targeting recruitment efforts at patients in the database with no insurance, or with Medicaid or Medicare without supplemental insurance. Patient enrollment demographics were monitored periodically so that subsequent recruitment mailings could be prioritized to ensure a balanced study population. Midway through enrollment, the proportion of men was significantly below goal; in response, mailings and recruitment calls were directed preferentially to men. Despite these efforts, Table 2 indicates that most study participants were at least high school-educated and reported adequate financial resources, and fewer than 40% were men.

The patient intervention required weekly attendance at group sessions that lasted up to 2 hours. Several of the clinics served rural areas requiring significant travel by the participants. Some communities were not accustomed to the research activities of AMCs and, thus, patients in those areas may have been less inclined to join a research study for reasons related to lack of trust or information. While these factors mimic challenges to implementation of the patient intervention in real-world settings, they created a greater challenge for recruitment into a research study, especially given that recruitment was based on blind mailings. The initial recruitment letter asked potential participants to call the study team only if they were not interested in participating (an opt-out strategy); if the study team did not receive a response, then a follow-up call was made to answer any questions and determine interest in participating. However, the ratio of calls to enrollees was low, so the recruitment letter was changed to allow patients to also opt in. This option allowed participants to self-select for inclusion, which may have selected more motivated individuals but is still likely to generalize to a real-world implementation of the study intervention, which would ultimately require voluntary participation.

Recruitment of CHAs was somewhat challenging. The initial goal in enlisting CHAs was to recruit people with "natural helper" personalities from the same communities as the target patient population. To that end, flyers were posted in participating clinics, and clinic staffs and community leaders were asked to suggest people from within their communities. The response to this recruitment effort was relatively modest. Although we were able to recruit an adequate number of CHAs for the study with a minimal budget, our selection pool was limited. Future studies incorporating the CHA element may consider including a budget for a greater advertisement effort and other strategies to enhance recruitment.

Challenges involving the physicians were also encountered. A few providers left their participating clinical practices during the course of the 18-month intervention. In this situation, the provider's patients who were enrolled in HIP were referred to another participating provider within the practice. Also, completion of CPM forms often competed with busy and varying clinic schedules, the demands of patient care, or an upcoming review by the Joint Commission on the Accreditation of Healthcare Organizations. To minimize the additional work needed for collecting the CPM data, non-carbon copy paper was used, so that the top portion was used for the clinic note and the bottom portion was used for data entry. This was a brief 1-page form–the top portion was designed so that non-MD personnel (e.g., a triage nurse) could fill out the vital signs and medical history, while the bottom portion was filled out by the provider stating the medication changes and counseling that occurred during the visit. The physician strategy was developed by the study investigators who were not fully aware of all the QI initiatives (ongoing or planned) within the practices. The CPM form, while short and simple, was viewed by the practice staff as an additional form of which to keep track, and they had to modify their usual procedures 1 day a month to collect these data. With the increased use of electronic medical records, future studies should be able to integrate the collection of information about medication changes and counseling within routine charting, although this would also remove a reminder system that might contribute to improved BP management.

Developing the training modules was challenging due to the availability of multiple hypertension guidelines [8,39-41]. The original proposal was written when JNC-6 guidelines were widely used; after the proposal was written and before it was funded, the JNC-7 guidelines were released. The training materials were modified to match the newer version. To promote completion of the modules, they were offered on the internet (allowing providers to complete them when time was available), and providers were given CME credit to provide additional incentive for the time spent.

Overall, implementation of the study within a community setting was facilitated by including the network director (RJD) as part of the study team. The director had worked with the practices and providers on previous studies, and so her pre-existing relationships aided recruitment and troubleshooting of operational issues. Finally, it is important to define rewards and compensation for community practices, not only in terms of financial reimbursement, but also in terms of CME opportunities (categories 1 and 2), acknowledgment in publications, and dissemination of study results.

## Conclusion

Hypertension affects approximately 29% of the adult U.S. population and remains a leading cause of heart disease, stroke, and kidney failure. Despite numerous effective treatments, only 53% of people with treated hypertension are at goal blood pressure. The HIP interventions offered an opportunity to significantly improve BP control and lower CVD risk in a broad cross-section of hypertensive patients from community primary care practices. The study design will allow an assessment of the effectiveness and cost of the physician and patient interventions separately, so that health care organizations may make informed decisions about implementation of 1 or both interventions in the context of local resources.

## Competing interests

The authors declare that they have no competing interests.

## Authors' contributions

RJD, WSY, WFO, DBM, GPS, KIP, PL, and LPS made substantial contributions to the conception and design of the study. RJD, WSY, DBM, GPS, KIP, PL, JDA, MP, HLM, BCB, WF, and LPS contributed to the acquisition, analysis, and/or interpretation of the data. RJD, WSY, DBM, GPS, KIP, PL, and LPS have been involved in the drafting and/or revising of the manuscript. RJD, WSY, WFO, DBM, GPS, KIP, PL, JDA, MP, HLM, BCB, WF, and LPS have given final approval for the manuscript to be published.

## Supplementary Material

Additional file 1 **Algorithm pocket card.** BP management algorithm–derived from the JNC-7 guidelines and formatted as a decision tree Click here for file

Additional file 2 **Clinical Performance Measure (CPM) form**. data form that was completed by the MD Click here for file
