# Supplementary material for: Hypertension Improvement Project (HIP): study protocol and implementation challenges
Source: Trials. 2009 Feb 26;10:13. doi: 10.1186/1745-6215-10-13 (PMC2654882; doi:10.1186/1745-6215-10-13)

Clinical Performance Measures Summary

If	Then
**Office visit**	Blood pressure measured*
**SBP<120 and DBP<80 (Normal)**	Recheck within 2 years
**SBP=120-139 or DBP=80-89 (Prehypertension)**	Recheck within 1 year & provide advice on LM therapy
**Pts with no DM or CKD**	Goal BP <140/<90
**SBP=140-159 or DBP=90-99 (Stage 1)**	
This is pt's initial visit and currently not receiving therapy for BP	Confirm within 2 months
Pt is not receiving BP therapy and returns for follow-up visit for elevated BP:	• Trial of LM for 6-12 month<br>• If goal not met then start medication<br>• HCTZ is part of regimen for most<br>• At least monthly f/u until goal BP is reached<br>• If BP not at goal, adjust meds
**SBP >160 or DBP >100 (Stage 2)**	
This is pt's initial visit and currently not receiving therapy for BP	Confirm within 1 month
Pt is not receiving BP therapy and returns for follow-up visit for elevated BP:	• LM therapy and Medication<br>• HCTZ is part of the regimen for most<br>• At least monthly f/u until goal BP is reached<br>• If BP not at goal, adjust meds
**SBP≥180 or DBP≥110**	Assess for symptoms, check for papilledema, and evaluate or treat within 1 week
**Pts with DM or CKD**	Goal BP <130/<80
**SBP <130 or DBP <80**	Recheck within 1 year & provide advice on LM therapy
**SBP=130-139 or DBP=80-89**	• Trial of LM for 3 month<br>• If goal not met then start medication<br>• ACE-I or ARB is part of regimen<br>• At least monthly f/u until goal BP is reached<br>• If BP not at goal, adjust meds
**SBP ≥140 or DBP ≥90**	• LM therapy and Medication<br>• ACE-I or ARB is part of regimen<br>• At least monthly f/u until goal BP is reached<br>• If BP not at goal, adjust meds
**Pt receiving LM therapy & BP medications:**	
BP still above goal at follow-up	• Increase dose, add another BP medication, or substitute a BP medication<br>• At least monthly f/l until goal BP is reached

Key: SBP = systolic blood pressure, DBP = diastolic blood pressure; DM = diabetes mellitus; CKD = chronic kidney diease; f/u = follow-up; LM = lifestyle modification; HTN = hypertension. Blood pressures are in mm Hg.

Average of two or more readings separated by 30 seconds. Measurements should occur after 5 minutes of rest, seated, with back and arm supported and without food, caffeine, smoking or exercise in preceding 30 minutes.

DPG# 05102

Effective Brief Counseling

- Ask about current diet and physical activity habits.
- Advise patient to follow the DASH dietary pattern.
- Assess readiness to change lifestyle habits.
- Assist in setting realistic goals and action plan.
- Arrange a follow up visit.

Algorithm for LM

Ask about current diet and physical activity.

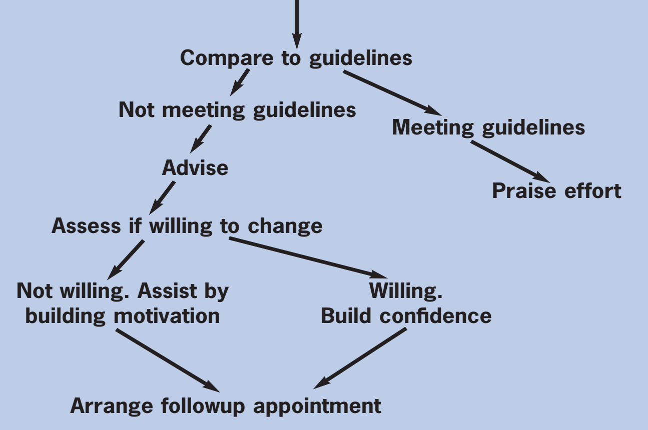

Prepared for the Hypertension Improvement Project
Sponsored by NHLBI/NIH Grant # 303-6276 Oct. 2004

Lifestyle Guidelines FOR Blood Pressure Control

Follow the DASH dietary pattern
(# servings for 2000 kcal diet)

- **4-5 servings Fruits/day**
  1 serving = 1 medium fruit, ½ cup, or 6 oz juice
- **4-5 servings Vegetables/day**
  1 serving = 1 cup raw, ½ cup cooked, or 6 oz juice
- **2-3 servings low-fat Dairy/day**
  1 serving = 8 oz milk,1 cup yogurt, or 1-½ oz cheese
- **7-8 servings Grains/day**
  1 serving = 1 slice bread, ½ cup rice, or pasta
- **2 or less servings Meats, Chicken & Fish/day**
  1 serving = 3 oz cooked, size of deck of cards
- **¼ serving Nuts, Seeds & Legumes/day**
  1 serving = ¼ cup nuts, 2 tbsp seeds, ½ cup beans
- **Limit fats and sweets**

And

- **Keep sodium intake to <2400 mg/day**
  (Read food label)
- **Lose weight if overweight**
  Overweight = BMI ≥25 kg/m^2
  Encourage weight loss at 2 lbs/wk.
- **Moderate alcohol intake**
  1 drink/day for women
  2 drinks/day for men
  1 drink = 12 oz beer, 5 oz wine or 1.5 oz liquor
- **Stay physically active**
  Moderate aerobic exercise 30 minutes/
  day most days of the week

HIP Hypertension Improvement Project

Blood Pressure Management for Patients Without DM or CKD [Goal <140/<90]

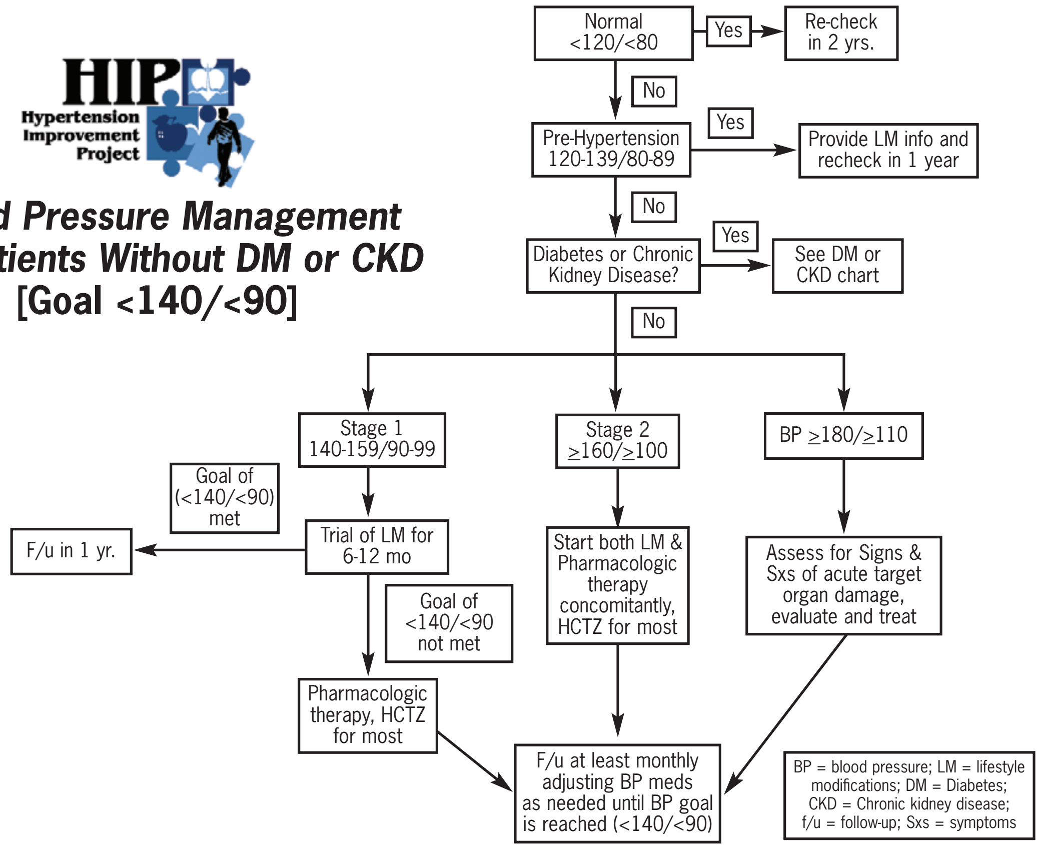

HIP Hypertension Improvement Project

Blood Pressure Management for Patients With DM or CKD [Goal <130/<80]

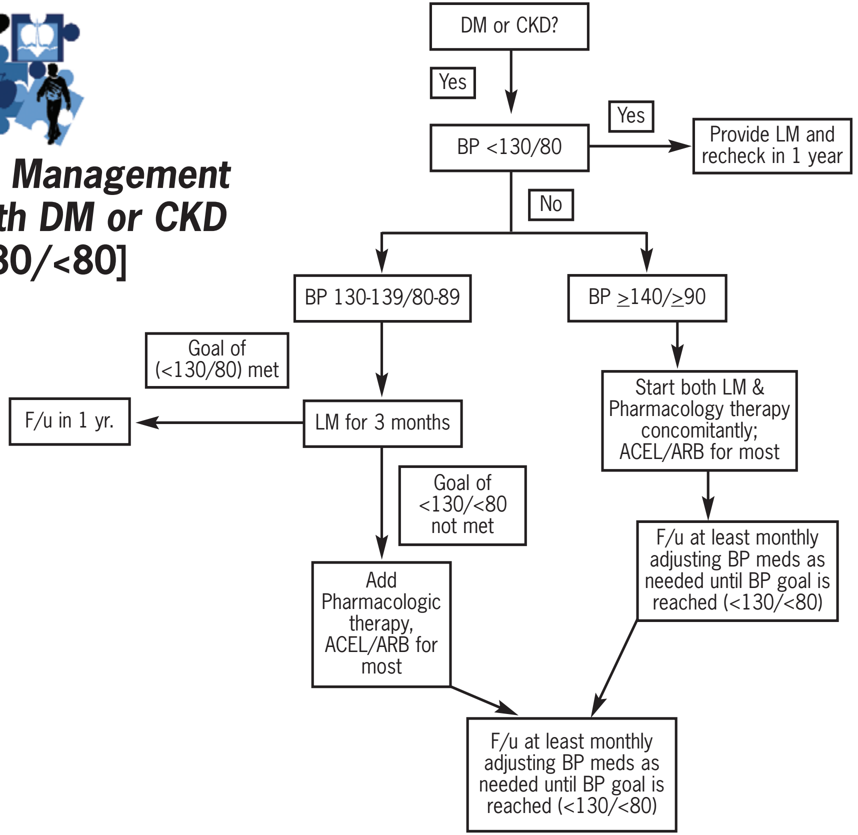

Supplement: Additional file 1 — Algorithm pocket card. BP management algorithm–derived from the JNC-7 guidelines and formatted as a decision tree [file 1745-6215-10-13-S1.pdf]
