# Supplementary material for: Hypertension Improvement Project (HIP): study protocol and implementation challenges
Source: Trials. 2009 Feb 26;10:13. doi: 10.1186/1745-6215-10-13 (PMC2654882; doi:10.1186/1745-6215-10-13)
Supplement: Additional file 2 — Clinical Performance Measure (CPM) form. data form that was completed by the MD [file 1745-6215-10-13-S2.pdf]

Provider ID #_____________

Study ID #:_____________

Clinical Performance Measure

Attach to encounter form at each patient visit.

TO BE COMPLETED BY NURSE OR PROVIDER

Patient Name: ______________________________

DOB: __/__/____ Duke MR # (If applicable)______________

Date of last visit: __/__/____ BP last visit: ___/___

Date of current visit: __/__/____ BP this visit: ___/___

Risk factors/co-morbidities (check all that apply):

__ Hypertension

__ Diabetes

__ Chronic Kidney Disease

__ Coronary disease

__ Heart failure

__ Obesity/Overweight (BMI ≥ 25)

__ CVD

Weight: ________lbs Height: _______inches

TO BE COMPLETED BY PROVIDER

Current BP medications at the start of this visit (check all that apply;check both classes if on combination drug):

___ Diuretic __Thiazide __ Other Diuretic

___ Beta blocker ___ Ace inhibitor

___ Calcium channel blocker ___ Angiotensin receptor blocker (ARB)

___ Peripheral alpha antagonist (e.g., doxazosin/cardura) ___ Other (please specify _________)

___ Central alpha agonists (e.g., clonidine)

___ Vasodilator (e.g., minoxidil, hydralazine)

Is this patient at goal BP? (General <140/90; DM/CKD <130/80) __yes __no

Actions taken on this visit (check all that apply):

___ Start new BP medication ___ Counsel for weight loss

___ Adjust dose of current BP medication ___ Counsel to reduce sodium intake

___ Stop BP medication ___ Counsel to increase physical activity

___ Refer to hypertension specialist ___ Counsel to reduce alcohol intake

(or to cardiologist or nephrologist for BP control) ___ Counsel on DASH diet

___ Refer for dietary counseling ___ Counsel regarding adherence

___ No action taken

Number of minutes spent addressing BP: ____ minutes

Time of next scheduled visit: __ weeks or __ months

Follow-up visit with ___MD ___Other health professional
